# Hydrogel Development for Rotator Cuff Repair

**DOI:** 10.3389/fbioe.2022.851660

**Published:** 2022-06-15

**Authors:** Zhengyu Xu, Yifei Fang, Yao Chen, Yushuang Zhao, Wei Wei, Chong Teng

**Affiliations:** ^1^ Department of Orthopaedics, The Fourth Affiliated Hospital, Zhejiang University School of Medicine, Yiwu, China; ^2^ Key Laboratory of Tissue Engineering and Regenerative Medicine of Zhejiang Province, Dr. Li Dak Sum & Yip Yio Chin Center for Stem Cells and Regenerative Medicine, Zhejiang University School of Medicine, Hangzhou, China

**Keywords:** hydrogel, rotator cuff, tissue engineering, regeneration medicine, cytokine

## Abstract

Rotator cuff tears (RCTs) are common in shoulder disease and disability. Despite significant advances in surgical repair techniques, 20–70% of patients still have postoperative rotator cuff dysfunction. These functional defects may be related to retear or rotator cuff quality deterioration due to tendon retraction and scar tissue at the repair site. As an effective delivery system, hydrogel scaffolds may improve the healing of RCTs and be a useful treatment for irreparable rotator cuff injuries. Although many studies have tested this hypothesis, most are limited to laboratory animal experiments. This review summarizes differences in hydrogel scaffold construction, active ingredients, and application methods in recent research. Efforts to determine the indications of hydrogel scaffolds (with different constructions and cargos) for various types of RCTs, as well as the effectiveness and reliability of application methods and devices, are also discussed.

## 1 Introduction

The rotator cuff is a complex of the supraspinatus, teres minor, infraspinatus, and subscapularis muscles and their tendons. They form a cuff-like structure around the humerus head and coordinate to complete complex shoulder movements (Craig et al., 2017). Rotator cuff injury and especially tear tends to occur with extrinsic factors such as age, trauma and strain, as well as intrinsic factors such as tendon degeneration, insufficient blood supply, and subacromial impingement (Nho et al., 2008; Yadav et al., 2009). This can lead to shoulder pain, reduced strength, and motion range restriction (Chakravarty and Webley, 1993; Colvin et al., 2012).

Rotator cuff tears (RCTs) require surgical repair, and outcomes have continuously improved with the development of medical techniques (Gurnani et al., 2015; Deprés-tremblay et al., 2016). Most patients have pain relief and improved shoulder function following surgery, but 20–70% complain about postoperative function (Tashjian et al., 2010; Koh et al., 2011; Toussaint et al., 2011). Some patients’ disability may be related to rotator cuff retear that appears as discontinuity on magnetic resonance imaging; however, patients with intact structures can still present dysfunction and weakness that may be related to tendon unit retraction, scar insertion, and adipose infiltration (Goutallier et al., 1994; Liem et al., 2007; Sugaya et al., 2007; Kuzel et al., 2013). Therefore, the “healing” concept of rotator cuff repair should be defined more prudently and include structural integrity and functionality.

**FIGURE 1:**
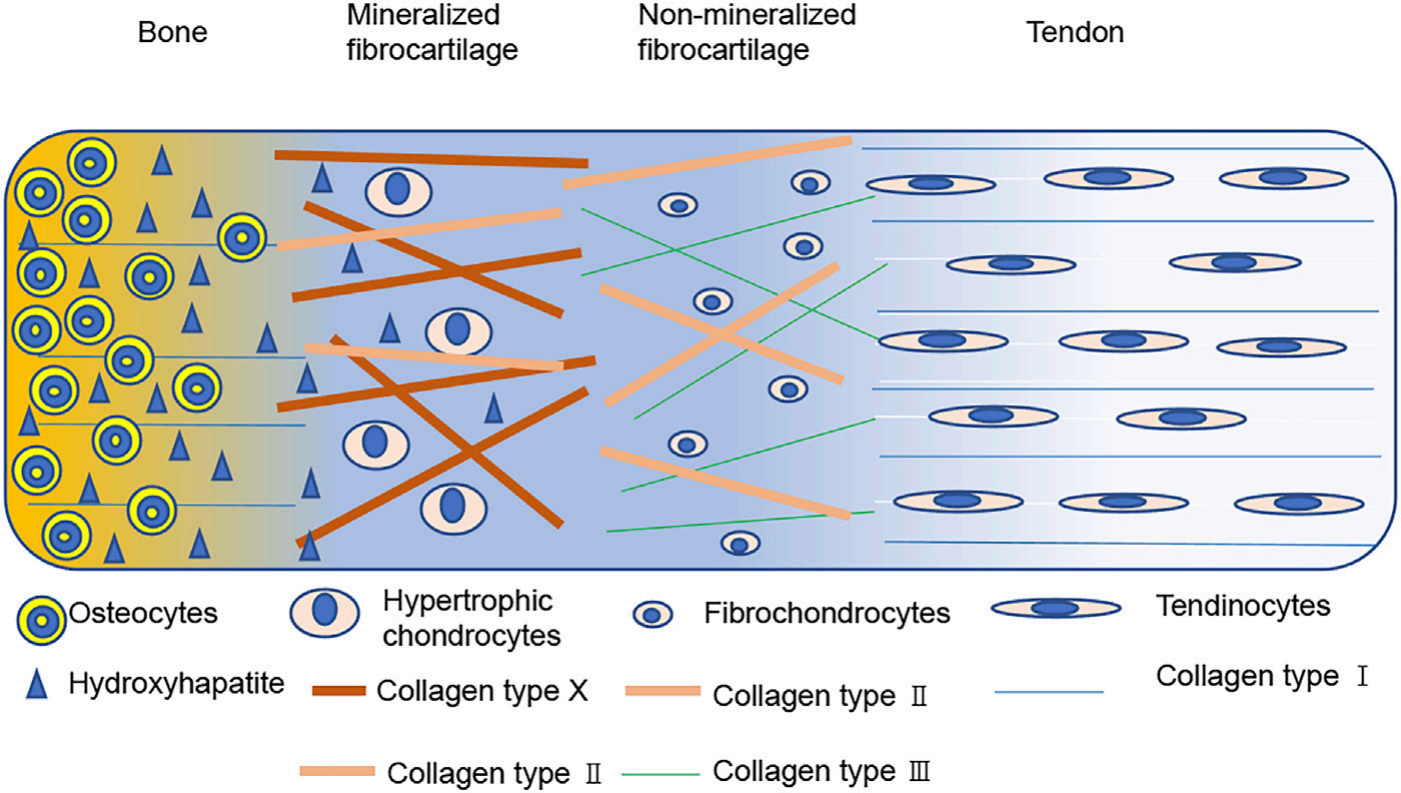
Diagram of the four zones at the bone-tendon interface.

Most RCTs occur at the tendon-bone interface where the surgery reconstruction takes place (Thorsness and Romeo, 2016; Oh et al., 2018), and this site is the key for rotator cuff healing (Li et al., 2019; Chae et al., 2020; Huang et al., 2020). For better understanding of the rotator cuff healing process, we need more knowledge about the histological structure. The tendon-bone interface is a gradient region that includes the tendon, uncalcified fibrocartilage, calcified fibrocartilage, and bone with different cells, structures, and mechanical aspects (Bonnevie and Mauck, 1545). The tendinocytes are distributed in the tendon tissue and contain type I collagen, while fibrochondrocytes are arranged along the long axis of collagen fibers and distributed in the non-mineralized fibrocartilage containing types I, II, and III collagen. Hypertrophic chondrocytes arranged in a columnar pattern exist in the mineralized fibrocartilage and contain type I, II, and X collagen. Finally, osteocytes, osteoblasts, and osteoclasts can be seen in bone and contain type I collagen (Figure 1). In addition, mineral contents and proteoglycan species vary among the different regions, and this natural heterogeneity results in varied mechanical and biological properties of the interface tissue, effectively reducing stress and allowing loads to be transferred from the tendon to the bone (Spalazzi et al., 1932; Rossetti et al., 1476).

Increasing attention has been paid to the application of interfacial tissue engineering for rotator cuff injury repair. Engineered interfacial tissue is mainly composed of seed cells, growth factors, and biomaterials. Seed cells provide the source of regeneration of the defect tissue through migration, adhesion, proliferation, and differentiation (Park et al., 2016; Liu et al., 2019). Growth factors regulate specific signaling pathways to affect a variety of cell behaviors (Prabhath et al., 2018; Han et al., 2020). Biomaterials serve as bridges that can provide a suitable growth environment for seed cells and growth factors to interact (Zhao et al., 2017; Chae et al., 2020; Veronesi et al., 2020).

Hydrogel is a 3D network structure of hydrophilic polymer chains (Oliva et al., 2017; Dimatteo et al., 2018). The polymer system of hydrogel scaffolds provides a good substrate with potential for cell transplantation and differentiation, endogenous regeneration, biological repair, and continuous delivery of growth factors and active substances (Ma et al., 2018; Tang et al., 2018; Marycz et al., 2019). In order to promote tendon-bone interface healing for rotator cuff repair, these hydrogel scaffolds should meet several requirements. First, they must reproduce the multi-regional structure of tissue interface as much as possible, including matrix composition, microstructure, and mechanical characteristics. Second, scaffolds can support the adhesion, proliferation, and differentiation of specific phenotypes of different stem cells or progenitor cells. Thirdly, scaffolds should be degradable at a rate is similar to the tissue regeneration rate to continue releasing physiological load. Finally, the scaffold design should also consider clinical use and match with the corresponding reconstructive surgery.

## 2 Classifications and Characterization of Hydrogels Applied in Rotator Cuff Repair

### 2.1 Classification of Hydrogels

Hydrogels is a big class of biomaterials and can be classified according to different bases. The first one is whether their source is natural or synthetic. Natural hydrogels are obtained from plants or animals consisting of proteins and polysaccharides. They usually have good biocompatibility and biodegradability but poor mechanical strength. In recent years, synthetic hydrogels have gradually replaced natural hydrogels as they have better water absorption capacity, greater strength, and longer service life. However, synthetic hydrogels are inferior to natural ones in biological recognition, intercellular response, and cell-induced remodeling. Nichol et al. invented a hydrogel called gelatin methacryloyl (GelMA) that is a hybrid consisting of gelatin and methacryloyl (Nichol et al., 2010). Its biocompatibility is much better than gelatin and is similar to collagen, but with better formability. In the repair of rotator cuff injury, it is often used as a therapeutic substance carrier in rotator cuff injury repair (Cao et al., 2020; Huang et al., 2021). Another hydrogel classification is based on polymer constituents. Homopolymer hydrogels are derived from a single species of hydrophilic polymer or copolymer. Multipolymers hydrogels consist of two independent polymers or interpenetrating polymer networks. Besides, hydrogels can be classified based on whether they could responsive to environmental stimuli such as temperature, pH values, light, ionic strength, and magnetic fields. For example, ultraviolet light can stimulate the curing reaction of GelMA and form a three-dimensional structure suitable for cell growth and differentiation with good strength that provides a suitable extracellular microenvironment for stem cells used in rotator cuff repair (Cao et al., 2020). The classification types are shown in Table 1.

**TABLE 1:** Hydrogel classification.

Source	Natural Synthetic	Collagen, Gelatin, Chitosan, Hyaluronic acid, N-isopropyl Acrylamide (PNIPAM), Polyethylene Glycol (PEG), PoloxamerEtc.
Preparation	Homopolymer	A single species of polymer or copolymer
	Multipolymer	Two independent crosslinked components
Response	Chemical	pH response, oxidant response, glucose response
	Physical	Temperature response, pressure response, light response
	Biochemical	Enzyme response, ligand response, antigen response

### 2.2 Characterization of Hydrogels Applied in Rotator Cuff Repair

The main characterizations of hydrogels include swelling, self-healing, degradation and biocompatibility abilities. The physicochemical properties of hydrogels must be considered for appropriate application in rotator cuff repair. Stem cell differentiation, the loading of active substances and cytokines, and mimicking the multilayer structure of tendon-bone interface are all critical issues. The characterization and properties of hydrogels applied for rotator cuff repair are summarized in Table 2.

**TABLE 2:** Overview of hydrogels applied for rotator cuff repair.

Hydrogel Name (Abbreviation)	Hydrogel Features/Advantages for Rotator Cuff Repair Engineering	Limitations	Refs
Gelatin hydrogel	Biodegradable, thermo-responsive, elastic, injectable	Poor mechanical properties faster degradation rate	Tokunaga et al. (2015a); Tokunaga et al. (2015b); Kabuto et al. (2015); Tokunaga et al. (2017)
Collagen hydrogel	Natural ECM protein, reasonable biomechanical properties, injectable	Limited number of functional groups for crosslinking	Hee et al. (2011); Jiang et al. (2020)
Fibrin hydrogel	Easy to be functionalized impressive stiffness, injectable	Immune response	Janmey et al. (2009); Rothrauff et al. (2019); Jiang et al. (2020)
Gelatin methacryloyl hydrogel (GelMA)	Self-sterilization, low cost, Photopolymerized, high compatibility, injectable	poor tissue adhesivity	Rothrauff et al. (2019); Cao et al. (2020); Huang et al. (2021)
Hyaluronic acid hydrogel (HA)	Biocompatible, biodegradable, noncytotoxic, nonimmunogenic	Do not support cell attachment	Lin et al. (2021)
Alginate hydrogel	Quick cross-linking, mechanically strong	Non-biodegradable and elicit immunological responses	Thankam et al. (2021)
Chitosan hydrogel	Therapeutic substance delivery capacity, injectable	Low solubility and high viscosity	Funakoshi et al. (2005)
Chitosan-4-thiobutylamidine hydrogel (CS-TBA)	Biocompatible, highly absorbent, injectable, structurally similar to natural ECM	Low solubility, high viscosity, difficult for preparation	Teng et al. (2021)
Human tendon-derived collagen hydrogel (tHG)	Thermo-responsive, injectable, type I collagen-rich	xenogeneic immune response	Kaizawa et al. (2019a); Kaizawa et al. (2019b)
Ion-based hydrogels	Anti-inflammatory	Rapid ion release rate	Chen et al. (2021); Yang et al. (2021)
Polyethylene glycol diacrylate (PEGDA)	Biocompatible, degradable, Easily manipulated, non-immunogenic, injectable	Limited microenvironment control, Poor toughness	Chen et al. (2011)
Polyvinyl alcohol (PVA)	Mechanically strong, MSC chondrogenic differentiation	Biologically inert	Thankam et al. (2021)
Poly-lactic-co-glycolic acid (PLGA)	Biocompatibility, easy handling, Similar mechanical properties with tendon	potential toxicity from dose dumping, inconsistent drug release and drug-polymer interactions	Moffat et al. (2009); Xie et al. (2010)

#### 2.2.1 Physicochemical Properties

Physicochemical properties such as swelling and stiffness are basic characteristics of hydrogels. Swelling property comes from hydrophilic groups such as hydroxyl and carboxyl groups in the polymer networks (Cui et al., 2019). The hydrogel is hydrated in water, allowing soluble molecules to enter the gel and remain stable, eventually reaching a state of equilibrium. Hydrogen bonds formed between hydrophilic groups and water molecules in the polymer chains can stabilize the hydrogel structure and effectively encapsulate active substances. In the case of rotator cuff injury, the hydrogel can be used to load cytokines, active substances, or even stem cells to promote rotator cuff healing. Hydrogel stiffness is also crucial for cell function and differentiation. Pelham and Wang first claimed that the stiffness of biomimetic extracellular matrix (ECM) molecules could be a key factor in regulating cell shape, motility, and spreading (Pelham and Wang, 1998). Self-healing hydrogel could fuse together after being broken into fragments due to new bonds spontaneously formed. Since rotator cuff repair surgery is mostly performed arthroscopically, the hydrogel must have good rheological and mechanical properties. Some hydrogels can be injected through a tube or syringe with no performance change (Patil et al., 2018).

#### 2.2.2 Biodegradation and Biocompatibility

The degradation process of hydrogel must meet biological requirements. *In vivo* degradation should be considered during hydrogel preparation. The ideal preparation method must meet basic requirements for *in vivo* use, including no introduction of small molecule cross-linking agents and mild cross-linking without toxic by-products, (Huebsch et al., 2010; Toh et al., 2012; Toda et al., 2016). In addition to the physicochemical properties and functions of existing hydrogels, residual functional groups (not involved in network crosslinking) can be used to imbue different hydrogel functions. These groups can be added directly to the hydrogel to avoid adding these substances to the cell culture medium. Despite these advantages, covalent bonding is complex and more likely to produce toxic small molecules. This is due to purification techniques and insufficient degradation of cross-linked groups, which must be taken into account when designing hydrogel precursors. In general, materials using physically linked thermo-gelling and cryo-gelling showed low cytotoxicity compared to traditional photopolymerized hydrogels (Park et al., 2016).

The biomechanical stability of hydrogels is critical for therapy cargo reasonable release. Natural hydrogel’s biomechanical stability is poorer than the synthetic biomaterial hydrogel for which is always damaged by cellular enzyme. However, there are also some studies to improve the biomechanical stability of hydrogels by modifying the hydrogels from natural sources. Shi et al. add bisphosphonates (BPs) into hyaluronan (HA) and they find it`s enzymatic degradation rate is lower than the control HA hydrogel *in vitro*. The mechanism may be that the covalent incorporation between BPs and HA increases the hydrogel`s stiffness compared to the unmodified HA hydrogel. (Shi et al., 2018). Lee et al. find that the hyaluronidase inhibition activity can be inhibited by TA (tannic acid) and the HA-TA hydrogels’ enzymatic stability is significantly increased when compared with the control group (Lee et al., 2018).

Natural polymers (chitosan, collagen, alginate) and synthetic polymers (polylactic acid [PLA], polylactic hydroxyacetic acid [PLGA]) have been widely used in drug delivery for their biocompatibility, mechanical properties, and ease of handling. Synthetic hydrogels are robust and provide stability, repeatability and acceptability of cells for microenvironments. Material modification can promote cell adhesion and differentiation and maintain multiple potentials. For example, amino acids and bromo groups can promote the transformation of adipose-derived stem/stromal cells (ADSCs) into bone cells and adipose cells, respectively (Benoit et al., 2008; Liu et al., 2013). Phenyl and sulfhydryl groups have the potential to promote ADSC differentiation into chondrocytes (Ingber, 2003). Excipients can be added by manipulating the functional groups of hydrogel precursors to guide cell behavior and differentiation.

#### 2.2.3 Mechanical Properties

As the delivery system for the therapy cells, hydrogels mechanical properties are the critical parameters which regulate mechanotransduction signal-mediated cellular behaviors (Sieminski et al., 2007; Huebsch and Mooney, 2009). For example, the cell behaviors were widely regulated by the substrate stiffness (Wen et al., 2014). Most type of cells benefit from stiffer substrates for more organized cytoskeletons. Mechanical stimulation can induce MSCs to differentiate into tendon-bone cell lineage which is critical for rotator cuff repair (Visser et al., 2015).

Hydrogel scaffold used for rotator cuff repair and augmentation should meet the native biomechanical properties. Kristen et al. report that their PLGA scaffold has the similar mechanical properties with the tendon. The tensile modulus of the unaligned and aligned scaffolds averaged 107 and 341 MPa, respectively, while the mean ultimate tensile strength ranging from 3.7 to 12.0 MPa (Moffat et al., 2009).

For mild rotator cuff lesion, patients prefer conservative treatment. Therefore, these injectable hydrogels as the carrier of therapy cargos and cells have a good prospect for clinical application. Natural injectable hydrogels including collagen, fibrin, gelatin, chitosan. They usually have good biocompatibility while limited in the mechanical prosperity and immune response (Table 2). Synthetic biomaterial hydrogels such as PEGDA have better tunability, almost no immune response and stronger mechanical properties.

## 3 The Functions of Hydrogel Used in Rotator Cuff Repair

**FIGURE 2:**
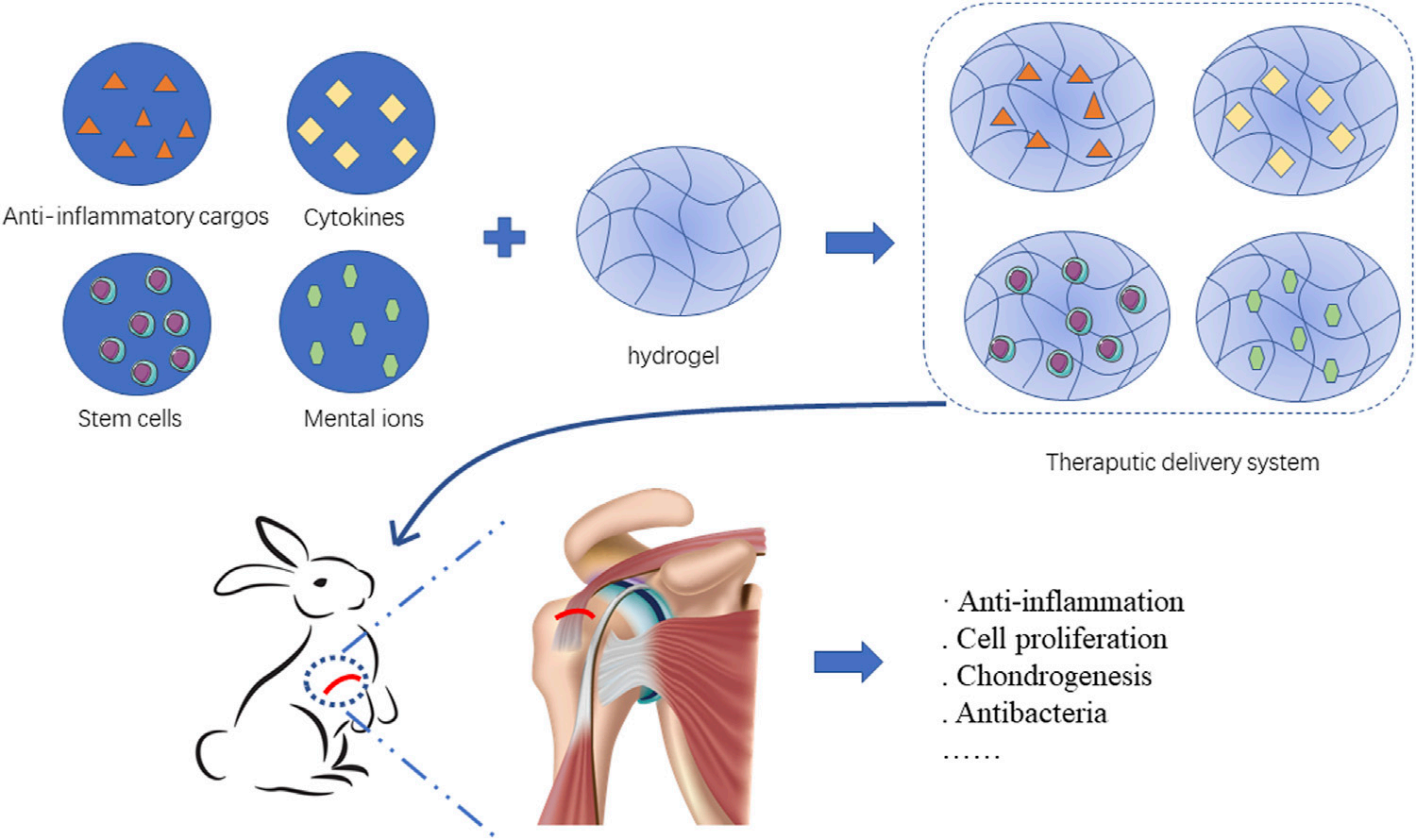
Hydrogels load therapeutic cargos such as Anti-inflammatory cargos, cytokines, stem cells and mental ions to facilitate rotator cuff healing.

Hydrogels can serve as delivery systems to carry multiple therapeutic components including Anti-inflammatory cargos, cytokines, stem cells and mental ions. These cargos benefit rotator cuff healing by different pattern such as anti-inflammation, cell proliferation, and chondrogenesis. (Figure 2).

### 3.1 Anti-Inflammatory Effects *via* its Therapeutic Cargo

Acute inflammation is the natural response in the early state of rotator cuff injury, while chronic inflammation is a major cause of rotator cuff quality deterioration, non-healing, or re-tearing after repair. Inhibiting chronic inflammation is an effective means to improve rotator cuff quality, promote healing, and prevent retear after surgical repair (Childress et al., 2013; Kuo et al., 1932). The hydrogel itself, such as HA or methacrylated collagen hydrogel or its therapeutic cargo, such as farnesol and curcumin, can be used to inhibit chronic inflammation after rotator cuff repair.

Matrix metalloproteinases (MMPs) are calcium and zinc dependent proteinases that break down ECM proteins. Elevated levels of metalloproteinases have been correlated with several inflammatory states including delayed healing wounds, malignant tumors and rotator cuff tears. In patients with massive rotator cuff tears, high levels of MMPs are found in the synovial fluid samples from glenohumeral joints (Jacob et al., 2012; Shih et al., 2018). With the goal of achieving inherent and long-term MMP regulation, Liang et al. developed an methacrylated collagen-HA hydrogel with controlled enzymatic degradability for MMP regulation. This novel strategy provides new insight into the hydrogel design for rotator cuff repair (Liang et al., 2018).

Farnesol is a sesquiterpene compound from fruits that exerts anti-inflammatory and antioxidative effects and promotes the synthesis of collagen. Lin et al. designed farnesol containing hydrogel membranes based on gellan gum and HA. The membranes could swell rapidly and adhere to the tear site, acting as a barrier and farnesol source during the repair period. Results indicated that farnesol enhanced collagen production and the hydrogel membranes was promising in the repair of rotator cuff injuries (Lin et al., 2021).

**FIGURE 3:**
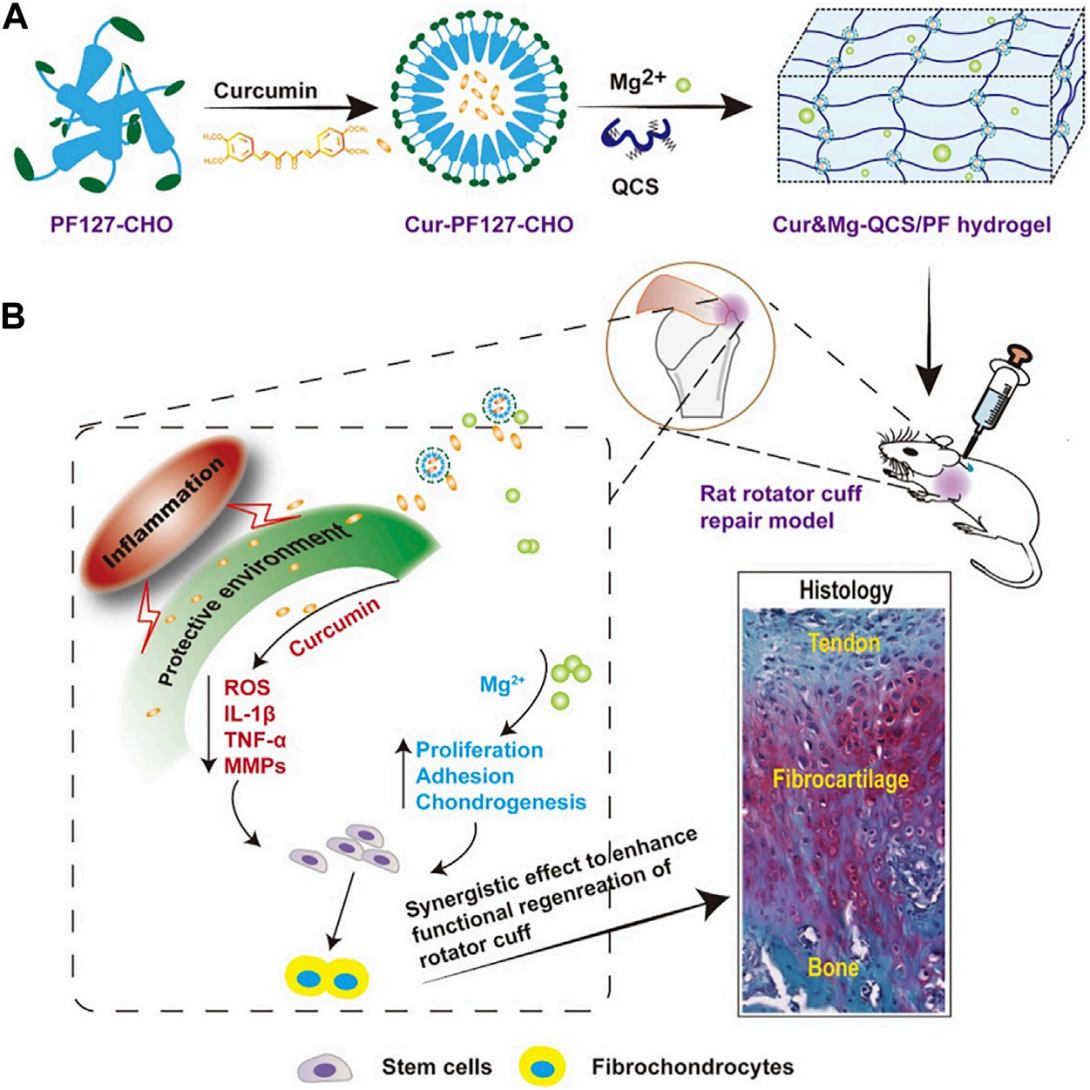
**(A)** Synthesis of Cur&Mg-QCS/PF hydrogels. **(B)** *In vivo* application and evaluation of Cur&Mg-QCS/PF hydrogels. (Reprinted from (Lin et al., 2021) with permission from Theranostics).

Curcumin is a natural compound with favorable anti-inflammatory properties. Chen et al. developed a novel hydrogel termed Cur&Mg-QCS/that could release curcumin in a controlled and highly efficient manner (Chen et al., 2021). The synthesis pathways is demonstrated in Figure 3A. Curcumin released at the repair site provided appropriate extracellular environment for stem cells by exerting antioxidative and anti-inflammatory effects to regulate levels of reactive oxygen species, IL-1β, TNF-α, and MMPs (Figure 3B).

### 3.2 Cytokine Delivery System

Cytokines are small peptides or glycoproteins produced by a variety of tissue cells. Cytokines include vascular endothelial growth factor (VEGF), transforming growth factor (TGF)-*β*, platelet-derived growth factor (PDGF), epidermal growth factor (EGF), and insulin-like growth factor (IGF). Cytokines can mediate cellular interactions and promote cell growth, collagen deposition, and angiogenesis to facilitate rotator cuff injury repair (Wu et al., 2017). Effectively transporting cytokines to the injury rotator cuff has become a research focus. The ideal delivery system should be easy to use, non-toxic, and biocompatible.

Arimura et al. confirmed that loading TGF-β1 with gelatin hydrogel inhibited MMP-9 and MMP-13 expression, thus increasing collagen accumulation and enhancing the formation of tough fibrous tissue at the healing site (Arimura et al., 2017). Fibroblast growth factor 2 (FGF-2) can improve rotator cuff healing after surgical repair. Tokunaga et al. found that the application of an FGF-2 impregnated gelatin hydrogel sheet (GHS) into the bone groove of the greater tubercles is conducive to the healing of rotator cuff in a rabbit model (Tokunaga et al., 2015b; Tokunaga et al., 2017). Previously, our group fabricated a dual-factor releasing sulfhydrylated chitosan hydrogel to deliver FGF-2 and Kartogenin (KGN) for the fast healing of the tendon-bone interface. KGN is a kind of small molecules which is believed to promote the chondrogenic differentiation of bone marrow-derived mesenchymal stem cells (BMSCs) (Johnson et al., 2012). KGN upgrades the expression of TGFβ1 while TGFβ1 stimulated cartilage nodule formation. Besides, KGN significantly increased the levels of phosphor-Smads that mediate TGFβ and BMP signaling (Decker et al., 2014). We found the FGF-2/KGN-loaded hydrogel could be a promising biomaterial to promote healing rotator cuff (Teng et al., 2021).

Bone morphogenetic protein (BMP)-7 promotes osteogenesis of chondrocytes and tendinocytes, as well as matrix formation. However, retaining local concentrations of BMP-7 is difficult due to its short half-life. Kabuto et al. studied continuous BMP-7 release using a GHS to stimulate the repair of rotator cuff at the tendon-bone insertion site (Kabuto et al., 2015). Tokunaga and colleagues used acidic GHS as a long-term delivery system for PDGF-BB. The PDGF-BB containing hydrogel attached supraspinatus tendon and induced superior collagen fiber orientation than the PDGF-BB free hydrogel (Tokunaga et al., 2015a).

Platelet-rich plasma (PRP) contains high concentrations of platelets obtained from the whole blood of animals or human after centrifugation. PRP also contains a variety of cytokines. Some groups have claimed that PRP has limited repair effect for rotator cuff injury, possibly because its release process is too rapid (Barber, 2018; Cavendish et al., 2020). Kim and colleagues designed a hydrogel loaded with PRP and self-assembled peptide (SAP) for RCT healing in a rat model.

SAP is an injectable hydrogel that can be injected into the body without surgery. The low toxicity and biodegradability make SAP become popular. Compared with traditional PRP injection, SAP has a nanofiber structure that can delay the release of factors contained in physiological environment which promote collagen production. They found that PRP can promote RCT healing by improving the collagen arrangement and inhibiting apoptosis and inflammatory changes (Kim et al., 2017).

### 3.3 Cellular Delivery System

As the seed of the tissue regeneration, stem cells have been clinically applied for over 20 years. The most common used stem cells for rotator cuff repair and regeneration are BMSCs, ADSCs, and tendon stem cells (Liu et al., 2013).

Hydrogels commonly used to deliver stem cells include tendon-derived collagen hydrogel (tHG), GelMa, and collagen. The mechanisms by which hydrogel with stem cells promotes rotator cuff healing include: 1) providing ECM materials to the damaged tendon, 2) hydrogel monomer functional groups can be modified to promote stem cell differentiation, 3) and hydrogel delivery systems can also deliver cytokines to promote stem cell differentiation (Toh et al., 2012; Park et al., 2016).

Cao et al. added osteoblasts, fibroblasts, and BMSCs separately in GelMA and sequentially loaded them on a 3D-printed multilayered scaffold to mimic the structure of tendon-bone interface. Chondrogenic differentiation was observed after *in vivo* implantation, suggesting that cells in a GelMA-multiphasic scaffold may be a new strategy to promote the healing of tendon-bone interface.

Kaizawa et al. invented a type I collagen-rich hydrogel based on human tendons, combining with ADSCs to improved mechanical strength of the tendon-bone interface (Kaizawa et al., 2019a; Kaizawa et al., 2019b). However, another group using the same model concluded that no biomechanical advantage was gained (Kaizawa et al., 2019a). Rothrauff et al. studied the effect of TGF-β3 and ADSCs delivered in fibrin and GelMA hydrogel on the healing after repairing acute or chronic massive RCTs in rats. They found the bone mineral density was improved with the application of fibrin, GelMA and ADSCs (Rothrauff et al., 2019).

Chen and colleagues developed an injectable hydrogel prepared by periosteal progenitor cells (PPCs) and polyethylene glycol diacrylate (PEGDA) with BMP-2 (Chen et al., 2011). They showed that BMP-2-loaded hydrogels could promote the differentiation of PPCs into osteoblasts and thus improve the success rate of tendon and bone healing. As a widely used biomaterial, PEGDA can provide a suitable microenvironment for mesenchymal stem cells (MSCs). In addition, bioactive cargos can be physically encapsulated in PEGDA hydrogels with MSCs as well.

### 3.4 Metal Ion Delivery System

Increasing attention has been paid to the role of metal ions in tendon-bone interface repair, including their anti-inflammatory, antibacterial, and cell differentiation-promoting abilities (Wang et al., 2021). Transporting metal ions and then slowly releasing them at the site of rotator cuff repair remain challenges.

**FIGURE 4:**
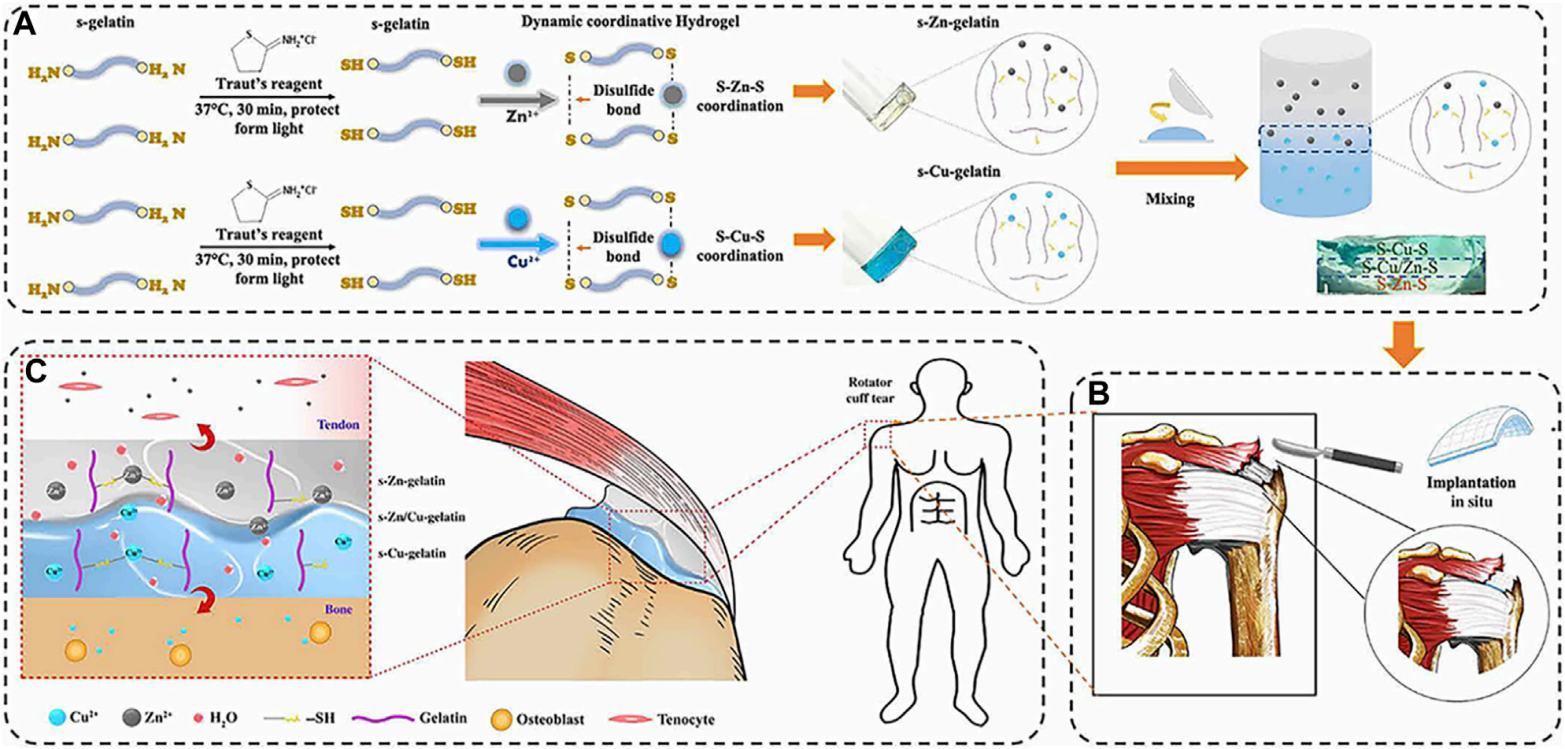
**(A)** The fabrication of gradient bimetallic hydrogels. **(B)** The application of the gradient bimetallic hydrogel for RCT. **(C)** The mechanism of gradient bimetallic hydrogel for the regeneration of tendon-bone interface. (Reprinted from (Yang et al., 2021) with permission from Science Advances).

Yang et al. constructed a gradient bimetal ion-based hydrogel for the first time by crosslinking sulfhydryl groups with zinc and copper ions for microstructural tendon-bone insertion reconstruction (Figure 4) (Yang et al., 2021). In this bimetallic hydrogel system, zinc and copper ions act as crosslinkers for the hydrogel and provide antibacterial effects and induce regeneration in the same time. The zinc and copper ions gradient layer were demonstrated to induce the arrangement of collagen and fibrocartilage at the tendon-bone interface. The gradient bimetallic ion-based hydrogels provide new insights into the regeneration of rotator cuff.

**FIGURE 5:**
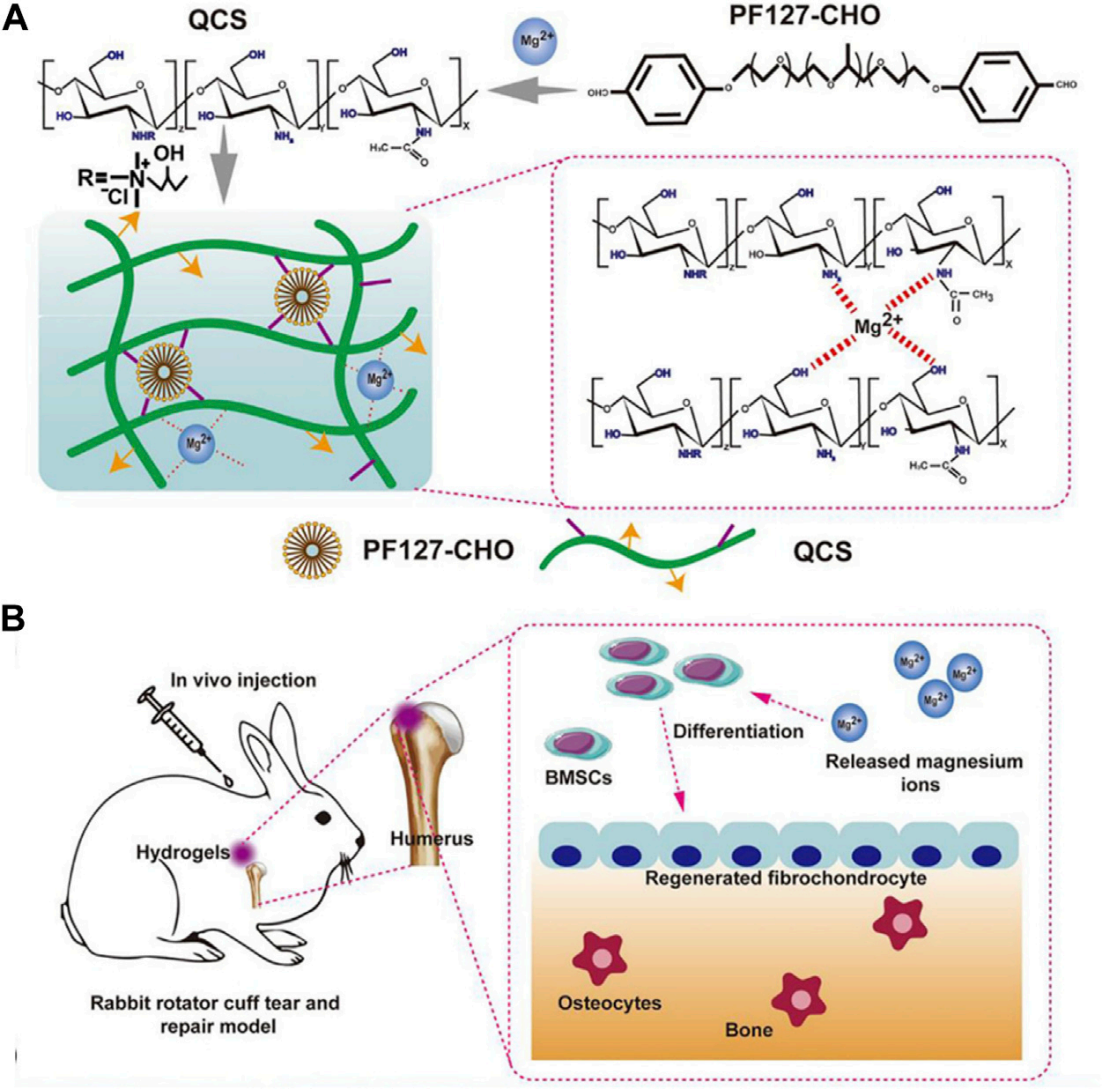
**(A)** Scheme of the fabrication of QCS/PF hydrogels and the interaction between Mg^2+^ and QCS; **(B)** Application of QCS/PF hydrogels delivering Mg^2+^ *in situ* to promote tendon-bone interface regeneration in the rabbit RCT model; (Reprinted from (Chen et al., 2020)with permission from Science Advances).

Magnesium ions promote cell adhesion, proliferation and fibrocartilage (Zhang et al., 2019; da Silva Lima et al., 2018; Hagandora et al., 2012). Besides, it can also regulate immune response which is essential for tendon-bone healing (Cheng et al., 2016). However, how to achieve the sustained release of magnesium ions is the key challenge. Chen et, al. reported a quaternized chitosan/Pluronic (QCS/PF) hydrogel delivering magnesium ions through metal coordination for RCT (Figure 5) (Chen et al., 2020). The self-healing property of this hydrogel ensured the safety of application in the RCT repair under external mechanical force, and the adhesive property increased the stability of material at the tendon-bone interface.

## 4 Multilayer Composite Hydrogel Scaffold for Rotator Cuff Repair

Jiang et al. developed a new method for RCT repair through combining a cell-laden collagen-fibrin hydrogel with a 3D printed PLGA scaffold (Jiang et al., 2020). This approach effectively supported human ADSCs’ proliferation and tenogenic differentiation. The innovation of this work lies in the good biocompatibility of PLGA scaffolds fabricated by 3D printing technology for rotator cuff tendon defect repair.

**FIGURE 6:**
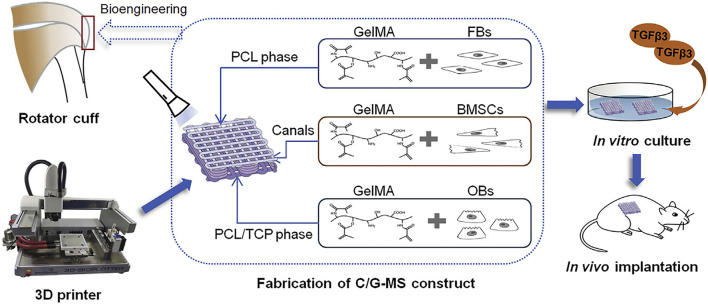
Illustration of 3D printing multiphasic scaffold for tendon-bone interface engineering. (Reprinted from (Cao et al., 2020) with permission from Elsevier).

Cao et al. used 3D printing technology to fabricate a multiphasic porous scaffold based on poly (ε-caprolactone) (PCL), PCL/tricalcium phosphate, and PCL/tricalcium phosphate. The three phases were designed to mimic the microstructure of tendon-bone interface. Osteoblasts, fibroblasts, and BMSCs and were separately encapsulated in GelMA hydrogel and sequentially loaded on the relevant scaffold phases (Figure 6). They found that 3D printing is an efficient method to develop multiphasic scaffold for tendon-bone interface engineering (Cao et al., 2020).

## 5 Conclusion and Outlook

RCTs are a common injury, and this repair is the most common shoulder operation. Tendon-bone healing following repair is influenced by the surgical technique, repair method, rotator cuff quality, and the biological healing process. This review covered hydrogels loaded with therapeutic cargoes such as cytokines, stem cells and bioactive materials that encourage rotator cuff healing. We recognize that this is a complex process dependent on numerous cellular signaling pathways involving different cytokines such as FGF-2, KGN, BMP-2, BMP-7, and PDGF-BB. Two recent studies have applied exosomes to rotator cuff injury; they can release a variety of cytokines that prevent inflammation and promote cell differentiation and fibrosis through a series of signaling pathways (Connor et al., 2019; Fu et al., 2021). Hydrogels loaded with exosomes for wound healing, bone regeneration, and cartilage repair have been studied (Hu et al., 2020; Wang et al., 2020; Zhao et al., 2020), but it is not clear whether they can promote rotator cuff healing, which may be worthy of investigation.

Besides tendon-bone healing, irreparable RCT management is another hot research topic. Surgeons encounter many challenges such as fatty infiltration, muscle atrophy, and tendon retraction in patients with irreparable RCT (Proctor, 2014; Lenart et al., 2015). Despite a substantial amount of research into rotator cuff patches, they have not applied been widely applied in the clinic. Patching the tendon-bone interface to promote rotator cuff-patch healing is difficult due to the complex gradual interface from bone tissue to tendon fiber tissue. Gradient layers of a composite hydrogel scaffold may be useful in this setting. These patches can experience stress in the early stage, but as cells migrate and the tendon fiber tissue grows, there is bionic healing between the bone and tendon.

The commercial application of hydrogel in wound dressing, drug delivery, and tissue engineering is quite successful. However, its application in shoulder is relatively rare. The possible factors are considered as follows: on the one hand, shoulder administration needs to be administered by injection or arthroscopy, which has high requirements on the physical and chemical properties of hydrogel or surgical techniques. Therefore, injectable hydrogels have great development potential. On the other hand, rotator cuff is consisted of tenacious tendons and construct the basic shoulder function. The hydrogel patch for rotator cuff tear need to achieve the similar toughness and strength. At present, the commercial application of some hydrogel materials to promote bone and cartilage repair provides ideas for the potential application in rotator cuff injury.

## References

[B1] Arimura H. Shukunami C. Tokunaga T. Karasugi T. Okamoto N. Taniwaki T. (2017). TGF-β1 Improves Biomechanical Strength by Extracellular Matrix Accumulation without Increasing the Number of Tenogenic Lineage Cells in a Rat Rotator Cuff Repair Model. Am. J. Sports Med. 45 (10), 2394–2404. 10.1177/0363546517707940 28586631

[B2] Barber F. A. (2018). PRP as an Adjunct to Rotator Cuff Tendon Repair. Sports Med. Arthrosc. Rev. 26 (2), 42–47. 10.1097/jsa.0000000000000193 29722762

[B3] Benoit D. S. W. Schwartz M. P. Durney A. R. Anseth K. S. (2008). Small Functional Groups for Controlled Differentiation of Hydrogel-Encapsulated Human Mesenchymal Stem Cells. Nat. Mater 7 (10), 816–823. 10.1038/nmat2269 18724374 PMC2929915

[B4] Bonnevie E. D. Mauck R. L. Physiology and Engineering of the Graded Interfaces of Musculoskeletal Junctions. Annu. Rev. Biomed. Eng. 20, 1545–4274. (Electronic)). 10.1146/annurev-bioeng-062117-121113 PMC7735380 29641907

[B5] Cao Y. Yang S. Zhao D. Li Y. Cheong S. S. Han D. (2020). Three-dimensional Printed Multiphasic Scaffolds with Stratified Cell-Laden Gelatin Methacrylate Hydrogels for Biomimetic Tendon-To-Bone Interface Engineering. J. Orthop. Transl. 23, 89–100. 10.1016/j.jot.2020.01.004 PMC7267011 32514393

[B6] Cavendish P. A. Everhart J. S. DiBartola A. C. Eikenberry A. D. Cvetanovich G. L. Flanigan D. C. (2020). The Effect of Perioperative Platelet-Rich Plasma Injections on Postoperative Failure Rates Following Rotator Cuff Repair: a Systematic Review with Meta-Analysis. J. shoulder Elb. Surg. 29 (5), 1059–1070. 10.1016/j.jse.2020.01.084 32305103

[B7] Chae S. Sun Y. Choi Y. J. Ha D. H. Jeon I. H. Cho D. W. (2020). 3D Cell-Printing of Tendon-Bone Interface Using Tissue-Derived Extracellular Matrix Bioinks for Chronic Rotator Cuff Repair. Biofabrication 13 (3), abd159. 10.1088/1758-5090/abd159 33285539

[B8] Chakravarty K. Webley M. (1993). Shoulder Joint Movement and its Relationship to Disability in the Elderly. J. Rheumatol. 20 (8), 1359–1361. 8230019

[B9] Chen B. Liang Y. Bai L. Xu M. Zhang J. Guo B. (2020). Sustained Release of Magnesium Ions Mediated by Injectable Self-Healing Adhesive Hydrogel Promotes Fibrocartilaginous Interface Regeneration in the Rabbit Rotator Cuff Tear Model. Chem. Eng. J. 396. 10.1016/j.cej.2020.125335

[B10] Chen B. Liang Y. Zhang J. Bai L. Xu M. Han Q. (2021). Synergistic Enhancement of Tendon-To-Bone Healing via Anti-inflammatory and Pro-differentiation Effects Caused by Sustained Release of Mg2+/curcumin from Injectable Self-Healing Hydrogels. Theranostics 11 (12), 5911–5925. 10.7150/thno.56266 33897889 PMC8058719

[B11] Chen C.-H. Chang C.-H. Wang K.-C. Su C.-I. Liu H.-T. Yu C.-M. (2011). Enhancement of Rotator Cuff Tendon-Bone Healing with Injectable Periosteum Progenitor Cells-BMP-2 Hydrogel *In Vivo* . Knee Surg. Sports Traumatol. Arthrosc. 19 (9), 1597–1607. 10.1007/s00167-010-1373-0 21327764

[B12] Cheng P. Han P. Zhao C. Zhang S. Wu H. Ni J. (2016). High-purity Magnesium Interference Screws Promote Fibrocartilaginous Entheses Regeneration in the Anterior Cruciate Ligament Reconstruction Rabbit Model via Accumulation of BMP-2 and VEGF. BIOMATERIALS 81, 14–26. 10.1016/j.biomaterials.2015.12.005 26713681

[B13] Childress M. A. Beutler A. Kuo L. A.-O. Chen H. M. Yu P. A. Chen C. L. (2013). Management of Chronic Tendon Injuries. Am. Fam. Physician 87 (7), 486–490. 23547590

[B14] Colvin A. C. Egorova N. Harrison A. K. Moskowitz A. Flatow E. L. (2012). National Trends in Rotator Cuff Repair. J. Bone Jt. Surgery-American Volume 94 (3), 227–233. 10.2106/jbjs.j.00739 PMC3262185 22298054

[B15] Connor D. E. Paulus J. A. Dabestani P. J. Thankam F. K. Dilisio M. F. Gross R. M. (2019). Therapeutic Potential of Exosomes in Rotator Cuff Tendon Healing. J. Bone Min. Metab. 37 (5), 759–767. 10.1007/s00774-019-01013-z PMC6830879 31154535

[B16] Craig R. Holt T. Rees J. L. (2017). Acute Rotator Cuff Tears. Bmj 359, j5366. 10.1136/bmj.j5366 29229593

[B17] Cui X. Lee J. J. L. Chen W. N. (2019). Eco-friendly and Biodegradable Cellulose Hydrogels Produced from Low Cost Okara: towards Non-toxic Flexible Electronics. Sci. Rep. 9, 18166. 10.1038/s41598-019-54638-5 31796821 PMC6890720

[B18] da Silva Lima F. da Rocha Romero A. B. Hastreiter A. Nogueira-Pedro A. Makiyama E. Colli C. (2018). An Insight into the Role of Magnesium in the Immunomodulatory Properties of Mesenchymal Stem Cells. J. Nutr. Biochem. 55, 200–208. 10.1016/j.jnutbio.2018.02.006 29554498

[B19] Decker R. S. Koyama E. Enomoto-Iwamoto M. Maye P. Rowe D. Zhu S. (2014). Mouse Limb Skeletal Growth and Synovial Joint Development Are Coordinately Enhanced by Kartogenin. Dev. Biol. 395 (2), 255–267. 10.1016/j.ydbio.2014.09.011 25238962 PMC4253021

[B20] Deprés-tremblay G. Chevrier A. Snow M. Hurtig M. B. Rodeo S. Buschmann M. D. (2016). Rotator Cuff Repair: a Review of Surgical Techniques, Animal Models, and New Technologies under Development. J. shoulder Elb. Surg. 25 (12), 2078–2085. 10.1016/j.jse.2016.06.009 27554609

[B21] Dimatteo R. Darling N. J. Segura T. (2018). *In Situ* forming Injectable Hydrogels for Drug Delivery and Wound Repair. Adv. Drug Deliv. Rev. 127, 167–184. 10.1016/j.addr.2018.03.007 29567395 PMC6003852

[B22] Fu G. Lu L. Pan Z. Fan A. Yin F. (2021). Adipose-derived Stem Cell Exosomes Facilitate Rotator Cuff Repair by Mediating Tendon-Derived Stem Cells. Regen. Med. 16 (4), 359–372. 10.2217/rme-2021-0004 33871287

[B23] Funakoshi T. Majima T. Iwasaki N. Suenaga N. Sawaguchi N. Shimode K. (2005). Application of Tissue Engineering Techniques for Rotator Cuff Regeneration Using a Chitosan-Based Hyaluronan Hybrid Fiber Scaffold. Am. J. Sports Med. 33 (8), 1193–1201. 10.1177/0363546504272689 16000663

[B24] Goutallier D. Postel J.-M. Bernageau J. Lavau L. Voisin M.-C. (1994). Fatty Muscle Degeneration in Cuff Ruptures. Clin. Orthop. Relat. Res. 304, 78–83. 10.1097/00003086-199407000-00014 8020238

[B25] Gurnani N. van Deurzen D. F. Flipsen M. Raven E. E. van den Bekerom M. P. (2015). Efficacy of Different Rotator Cuff Repair Techniques. Surg. Technol. Int. 26, 295–300. 26055023

[B26] Hagandora C. K. Tudares M. A. Almarza A. J. (2012). The Effect of Magnesium Ion Concentration on the Fibrocartilage Regeneration Potential of Goat Costal Chondrocytes. Ann. Biomed. Eng. 40 (3), 688–696. 10.1007/s10439-011-0433-z 22009314

[B27] Han B. Jones I. A. Yang Z. Fang W. Vangsness C. T. Jr. (2020). Repair of Rotator Cuff Tendon Defects in Aged Rats Using a Growth Factor Injectable Gel Scaffold. Arthrosc. J. Arthrosc. Relat. Surg. 36 (3), 629–637. 10.1016/j.arthro.2019.09.015 31784364

[B28] Hee C. K. Dines J. S. Dines D. M. Roden C. M. Wisner-Lynch L. A. Turner A. S. (2011). Augmentation of a Rotator Cuff Suture Repair Using rhPDGF-BB and a Type I Bovine Collagen Matrix in an Ovine Model. Am. J. Sports Med. 39 (8), 1630–1640. 10.1177/0363546511404942 21555508

[B29] Hu H. Dong L. Bu Z. Shen Y. Luo J. Zhang H. (2020). miR‐23a‐3p‐abundant Small Extracellular Vesicles Released from Gelma/nanoclay Hydrogel for Cartilage Regeneration. J. Extracell. vesicles 9 (1), 1778883. 10.1080/20013078.2020.1778883 32939233 PMC7480606

[B30] Huang C. Zhang X. Luo H. Pan J. Cui W. Cheng B. (2021). Effect of Kartogenin-Loaded Gelatin Methacryloyl Hydrogel Scaffold with Bone Marrow Stimulation for Enthesis Healing in Rotator Cuff Repair. J. shoulder Elb. Surg. 30 (3), 544–553. 10.1016/j.jse.2020.06.013 32650072

[B31] Huang Y. He B. Wang L. Yuan B. Shu H. Zhang F. (2020). Bone Marrow Mesenchymal Stem Cell-Derived Exosomes Promote Rotator Cuff Tendon-Bone Healing by Promoting Angiogenesis and Regulating M1 Macrophages in Rats. Stem Cell. Res. Ther. 11 (1), 496. 10.1186/s13287-020-02005-x 33239091 PMC7687785

[B32] Huebsch N. Arany P. R. Mao A. S. Shvartsman D. Ali O. A. Bencherif S. A. (2010). Harnessing Traction-Mediated Manipulation of the Cell/matrix Interface to Control Stem-Cell Fate. Nat. Mater 9 (6), 518–526. 10.1038/nmat2732 20418863 PMC2919753

[B33] Huebsch N. Mooney D. J. (2009). Inspiration and Application in the Evolution of Biomaterials. NATURE 462 (7272), 426–432. 10.1038/nature08601 19940912 PMC2848528

[B34] Ingber D. E. (2003). Tensegrity I. Cell Structure and Hierarchical Systems Biology. J. Cell. Sci. 116 (7), 1157–1173. 10.1242/jcs.00359 12615960

[B35] Jacob J. Eisemon E. Sheibani-Rad S. Patel A. Jacob T. Choueka J. (2012). Matrix Metalloproteinase Levels as a Marker for Rotator Cuff Tears. Orthopedics 35 (4), e474–8. 10.3928/01477447-20120327-18 22495845

[B36] Janmey P. A. Winer J. P. Weisel J. W. (2009). Fibrin Gels and Their Clinical and Bioengineering Applications. J. R. Soc. Interface. 6 (30), 1–10. 10.1098/rsif.2008.0327 18801715 PMC2575398

[B37] Jiang X. Wu S. Kuss M. Kong Y. Shi W. Streubel P. N. (2020). 3D Printing of Multilayered Scaffolds for Rotator Cuff Tendon Regeneration. Bioact. Mater. 5 (3), 636–643. 10.1016/j.bioactmat.2020.04.017 32405578 PMC7212184

[B38] Johnson K. Zhu S. Tremblay M. S. Payette J. N. Wang J. Bouchez L. C. (2012). A Stem Cell-Based Approach to Cartilage Repair. Science 336 (6082), 717–721. 10.1126/science.1215157 22491093

[B39] Spalazzi J. P. Boskey Al Fau - Pleshko N. Fau - Lu H. H. Pleshko. N. Lu H. H., Quantitative Mapping of Matrix Content and Distribution across the Ligament-To-Bone Insertion, (1932) 8(9):e74349 (Electronic)). 10.1371/journal.pone.0074349 PMC3760865 24019964

[B40] Kabuto Y. Morihara T. Sukenari T. Kida Y. Oda R. Arai Y. (2015). Stimulation of Rotator Cuff Repair by Sustained Release of Bone Morphogenetic Protein-7 Using a Gelatin Hydrogel Sheet. Tissue Eng. Part A 21 (13-14), 2025–2033. 10.1089/ten.TEA.2014.0541 25819324 PMC4507128

[B41] Kaizawa Y. Leyden J. Behn A. W. Tulu U. S. Franklin A. Wang Z. (2019). Human Tendon-Derived Collagen Hydrogel Significantly Improves Biomechanical Properties of the Tendon-Bone Interface in a Chronic Rotator Cuff Injury Model. J. Hand Surg. Am. 44 (10), 899–e11. 10.1016/j.jhsa.2018.11.021 30685142

[B42] Kaizawa Y. Franklin A. Leyden J. Behn A. W. Tulu U. S. Sotelo Leon D. (2019). Augmentation of Chronic Rotator Cuff Healing Using Adipose‐derived Stem Cell‐seeded Human Tendon‐derived Hydrogel. J. Orthop. Res. 37 (4), 877–886. 10.1002/jor.24250 30747435

[B43] Kim S. J. Lee S. M. Kim J. E. Kim S. H. Jung Y. (2017). Effect of Platelet-Rich Plasma with Self-Assembled Peptide on the Rotator Cuff Tear Model in Rat. J. Tissue Eng. Regen. Med. 11 (1), 77–85. 10.1002/term.1984 25643855

[B44] Koh K. H. Kang K. C. Lim T. K. Shon M. S. Yoo J. C. (2011). Prospective Randomized Clinical Trial of Single- versus Double-Row Suture Anchor Repair in 2- to 4-cm Rotator Cuff Tears: Clinical and Magnetic Resonance Imaging Results. Arthrosc. J. Arthrosc. Relat. Surg. 27 (4), 453–462. 10.1016/j.arthro.2010.11.059 21444007

[B45] Kuo L. A.-O. Chen H. M. Yu P. A. Chen C. L. Hsu W. H. Tsai Y. H. (1932). Depression Increases the Risk of Rotator Cuff Tear and Rotator Cuff Repair Surgery: A Nationwide Population-Based Study. PLoS One 14 (11), e0225778–6203. (Electronic)). 10.1371/journal.pone.0225778 PMC6876882 31765424

[B46] Kuzel B. R. Grindel S. Papandrea R. Ziegler D. (2013). Fatty Infiltration and Rotator Cuff Atrophy. J. Am. Acad. Orthop. Surg. 21 (10), 613–623. 10.5435/jaaos-21-10-613 24084435

[B47] Lee H.-Y. Hwang C.-H. Kim H.-E. Jeong S.-H. (2018). Enhancement of Bio-Stability and Mechanical Properties of Hyaluronic Acid Hydrogels by Tannic Acid Treatment. Carbohydr. Polym. 186, 290–298. 10.1016/j.carbpol.2018.01.056 29455990

[B48] Lenart B. A. Martens K. A. Kearns K. A. Gillespie R. J. Zoga A. C. Williams G. R. (2015). Treatment of Massive and Recurrent Rotator Cuff Tears Augmented with a Poly-L-Lactide Graft, a Preliminary Study. J. shoulder Elb. Surg. 24 (6), 915–921. 10.1016/j.jse.2014.09.044 25483907

[B49] Li H. Chen Y. Chen S. (2019). Enhancement of Rotator Cuff Tendon-Bone Healing Using Bone Marrow-Stimulating Technique along with Hyaluronic Acid. J. Orthop. Transl. 17, 96–102. 10.1016/j.jot.2019.01.001 PMC6551361 31194057

[B50] Liang H. Russell S. J. Wood D. J. Tronci G. (2018). A Hydroxamic Acid-Methacrylated Collagen Conjugate for the Modulation of Inflammation-Related MMP Upregulation. J. Mat. Chem. B 6 (22), 3703–3715. 10.1039/c7tb03035e 32254833

[B51] Liem D. Lichtenberg S. Magosch P. Habermeyer P. (2007). Magnetic Resonance Imaging of Arthroscopic Supraspinatus Tendon Repair. J. Bone Jt. Surgery-American Volume 89 (8), 1770–1776. 10.2106/00004623-200708000-00015 17671017

[B52] Lin Y. H. Lee S. I. Lin F. H. Wu G. X. Wu C. S. Kuo S. M. (2021). Enhancement of Rotator Cuff Healing with Farnesol-Impregnated Gellan Gum/Hyaluronic Acid Hydrogel Membranes in a Rabbit Model. Pharmaceutics 13 (7). 10.3390/pharmaceutics13070944 PMC8309098 34202556

[B53] Liu Q. Yu Y. Reisdorf R. L. Qi J. Lu C.-K. Berglund L. J. (2019). Engineered Tendon-Fibrocartilage-Bone Composite and Bone Marrow-Derived Mesenchymal Stem Cell Sheet Augmentation Promotes Rotator Cuff Healing in a Non-weight-bearing Canine Model. Biomaterials 192, 189–198. 10.1016/j.biomaterials.2018.10.037 30453215

[B54] Liu X. He J. Zhang S. Wang X. M. Liu H. Y. Cui F. Z. (2013). Adipose Stem Cells Controlled by Surface Chemistry. J. Tissue Eng. Regen. Med. 7 (2), 112–117. 10.1002/term.498 22162249

[B55] Ma Y. Lin M. Huang G. Li Y. Wang S. Bai G. (2018). 3D Spatiotemporal Mechanical Microenvironment: A Hydrogel-Based Platform for Guiding Stem Cell Fate. Adv. Mater 30 (49), e1705911. 10.1002/adma.201705911 30063260

[B56] Marycz K. Smieszek A. Trynda J. Sobierajska P. Targonska S. Grosman L. (2019). Nanocrystalline Hydroxyapatite Loaded with Resveratrol in Colloidal Suspension Improves Viability, Metabolic Activity and Mitochondrial Potential in Human Adipose-Derived Mesenchymal Stromal Stem Cells (hASCs). Polym. (Basel) 11 (1). 10.3390/polym11010092 PMC6402024 30960076

[B57] Moffat K. L. Kwei A. S.-P. Spalazzi J. P. Doty S. B. Levine W. N. Lu H. H. (2009). Novel Nanofiber-Based Scaffold for Rotator Cuff Repair and Augmentation. TISSUE Eng. PART A 15 (1), 115–126. 10.1089/ten.tea.2008.0014 18788982 PMC2809655

[B58] Nho S. J. Yadav H. Shindle M. K. Macgillivray J. D. (2008). Rotator Cuff Degeneration. Am. J. Sports Med. 36 (5), 987–993. 10.1177/0363546508317344 18413681

[B59] Nichol J. W. Koshy S. T. Bae H. Hwang C. M. Yamanlar S. Khademhosseini A. (2010). Cell-laden Microengineered Gelatin Methacrylate Hydrogels. Biomaterials 31 (21), 5536–5544. 10.1016/j.biomaterials.2010.03.064 20417964 PMC2878615

[B60] Oh J. H. Park M. S. Rhee S. M. (2018). Treatment Strategy for Irreparable Rotator Cuff Tears. Clin. Orthop. Surg. 10 (2), 119–134. 10.4055/cios.2018.10.2.119 29854334 PMC5964259

[B61] Oliva N. Conde J. Wang K. Artzi N. (2017). Designing Hydrogels for On-Demand Therapy. Acc. Chem. Res. 50 (4), 669–679. 10.1021/acs.accounts.6b00536 28301139 PMC6527116

[B62] Park H.-J. Jin Y. Shin J. Yang K. Lee C. Yang H. S. (2016). Catechol-functionalized Hyaluronic Acid Hydrogels Enhance Angiogenesis and Osteogenesis of Human Adipose-Derived Stem Cells in Critical Tissue Defects. Biomacromolecules 17 (6), 1939–1948. 10.1021/acs.biomac.5b01670 27112904

[B63] Patil S. Nune K. Misra R. (2018). Alginate/poly(amidoamine) Injectable Hybrid Hydrogel for Cell Delivery. J. Biomater. Appl. 33 (2), 295–314. 10.1177/0885328218790211 30096996

[B64] Pelham R. J. Wang Y.-L. (1998). Cell Locomotion and Focal Adhesions Are Regulated by Substrate Flexibility. Proc. Natl. Acad. Sci. U. S. A. 94 (25), 13661–13665. 10.1073/pnas.94.25.13661 PMC28362 9391082

[B65] Prabhath A. Vernekar V. N. Sanchez E. Laurencin C. T. (2018). Growth Factor Delivery Strategies for Rotator Cuff Repair and Regeneration. Int. J. Pharm. 544 (2), 358–371. 10.1016/j.ijpharm.2018.01.006 29317260 PMC8215558

[B66] Proctor C. S. (2014). Long-term Successful Arthroscopic Repair of Large and Massive Rotator Cuff Tears with a Functional and Degradable Reinforcement Device. J. shoulder Elb. Surg. 23 (10), 1508–1513. 10.1016/j.jse.2014.01.010 24725892

[B67] Rossetti L. Kuntz L. A. Kunold E. Schock J. Müller K. W. Grabmayr H. The Microstructure and Micromechanics of the Tendon-Bone Insertion. Nat. Mater 16, 1476–1122. (Print)). 10.1038/nmat4863 28250445

[B68] Rothrauff B. B. Smith C. A. Ferrer G. A. Novaretti J. V. Pauyo T. Chao T. (2019). The Effect of Adipose-Derived Stem Cells on Enthesis Healing after Repair of Acute and Chronic Massive Rotator Cuff Tears in Rats. J. shoulder Elb. Surg. 28 (4), 654–664. 10.1016/j.jse.2018.08.044 30527883

[B69] Shi L. Zhang Y. Ossipov D. (2018). Enzymatic Degradation of Hyaluronan Hydrogels with Different Capacity for *In Situ* Bio-Mineralization. Biopolymers 109 (2). 10.1002/bip.23090 29178472

[B70] Shih C.-A. Wu K.-C. Shao C.-J. Chern T.-C. Su W.-R. Wu P.-T. (2018). Synovial Fluid Biomarkers: Association with Chronic Rotator Cuff Tear Severity and Pain. J. shoulder Elb. Surg. 27 (3), 545–552. 10.1016/j.jse.2017.09.020 29169956

[B71] Sieminski A. L. Was A. S. Kim G. Gong H. Kamm R. D. (2007). The Stiffness of Three-Dimensional Ionic Self-Assembling Peptide Gels Affects the Extent of Capillary-like Network Formation. Cell. Biochem. Biophys. 49 (2), 73–83. 10.1007/s12013-007-0046-1 17906362

[B72] Sugaya H. Maeda K. Matsuki K. Moriishi a. J. (2007). Repair Integrity and Functional Outcome after Arthroscopic Double-Row Rotator Cuff Repair. J. Bone Jt. Surgery-American Volume 89 (5), 953–960. 10.2106/00004623-200705000-00006 17473131

[B73] Tang J.-N. Cores J. Huang K. Cui X.-L. Luo L. Zhang J.-Y. (2018). Concise Review: Is Cardiac Cell Therapy Dead? Embarrassing Trial Outcomes and New Directions for the Future. STEM CELLS Transl. Med. 7 (4), 354–359. 10.1002/sctm.17-0196 29468830 PMC5866934

[B74] Tashjian R. Z. Hollins A. M. Kim H.-M. Teefey S. A. Middleton W. D. Steger-May K. (2010). Factors Affecting Healing Rates after Arthroscopic Double-Row Rotator Cuff Repair. Am. J. Sports Med. 38 (12), 2435–2442. 10.1177/0363546510382835 21030564

[B75] Teng C. Fang Y. Zhu H. Huang L. Jin Y. Ye Z. (2021). A Dual-Factor Releasing Hydrogel for Rotator Cuff Injury Repair. Front. Mater. 8. 10.3389/fmats.2021.754973

[B76] Thankam F. G. Diaz C. Chandra I. Link J. Newton J. Dilisio M. F. (2021). Hybrid Interpenetrating Hydrogel Network Favoring the Bidirectional Migration of Tenocytes for Rotator Cuff Tendon Regeneration. J. Biomed. Mater Res. B Appl. Biomater. 110 (2), 467–477. 10.1002/jbm.b.34924 34342931

[B77] Thorsness R. Romeo A. (2016). Massive Rotator Cuff Tears: Trends in Surgical Management. Orthopedics 39 (3), 145–151. 10.3928/01477447-20160503-07 27214881

[B78] Toda H. Yamamoto M. Uyama H. Tabata Y. (2016). Fabrication of Hydrogels with Elasticity Changed by Alkaline Phosphatase for Stem Cell Culture. Acta biomater. 29, 215–227. 10.1016/j.actbio.2015.10.036 26525116

[B79] Toh W. S. Lim T. C. Kurisawa M. Spector M. (2012). Modulation of Mesenchymal Stem Cell Chondrogenesis in a Tunable Hyaluronic Acid Hydrogel Microenvironment. Biomaterials 33 (15), 3835–3845. 10.1016/j.biomaterials.2012.01.065 22369963

[B80] Tokunaga T. Ide J. Arimura H. Nakamura T. Uehara Y. Sakamoto H. (2015). Local Application of Gelatin Hydrogel Sheets Impregnated with Platelet-Derived Growth Factor BB Promotes Tendon-To-Bone Healing after Rotator Cuff Repair in Rats. Arthrosc. J. Arthrosc. Relat. Surg. 31 (8), 1482–1491. 10.1016/j.arthro.2015.03.008 25911389

[B81] Tokunaga T. Karasugi T. Arimura H. Yonemitsu R. Sakamoto H. Ide J. (2017). Enhancement of Rotator Cuff Tendon-Bone Healing with Fibroblast Growth Factor 2 Impregnated in Gelatin Hydrogel Sheets in a Rabbit Model. J. shoulder Elb. Surg. 26 (10), 1708–1717. 10.1016/j.jse.2017.03.020 28506489

[B82] Tokunaga T. Shukunami C. Okamoto N. Taniwaki T. Oka K. Sakamoto H. (2015). FGF-2 Stimulates the Growth of Tenogenic Progenitor Cells to Facilitate the Generation of Tenomodulin-Positive Tenocytes in a Rat Rotator Cuff Healing Model. Am. J. Sports Med. 43 (10), 2411–2422. 10.1177/0363546515597488 26311443

[B83] Toussaint B. Schnaser E. Bosley J. Lefebvre Y. Gobezie R. (2011). Early Structural and Functional Outcomes for Arthroscopic Double-Row Transosseous-Equivalent Rotator Cuff Repair. Am. J. Sports Med. 39 (6), 1217–1225. 10.1177/0363546510397725 21427446

[B84] Veronesi F. Borsari V. Contartese D. Xian J. Baldini N. Fini M. (2020). The Clinical Strategies for Tendon Repair with Biomaterials: A Review on Rotator Cuff and Achilles Tendons. J. Biomed. Mater Res. 108 (5), 1826–1843. 10.1002/jbm.b.34525 31785081

[B85] Visser J. Levett P. A. te Moller N. C. Besems J. Boere K. W. van Rijen M. H. (2015). Crosslinkable Hydrogels Derived from Cartilage, Meniscus, and Tendon Tissue. Tissue Eng. Part A 21 (7-8), 1195–1206. 10.1089/ten.TEA.2014.0362 25557049 PMC4394887

[B86] Wang J. Xu J. Wang X. Sheng L. Zheng L. Song B. (2021). Magnesium-pretreated Periosteum for Promoting Bone-Tendon Healing after Anterior Cruciate Ligament Reconstruction. Biomaterials 268, 120576. 10.1016/j.biomaterials.2020.120576 33271449

[B87] Wang L. Wang J. Zhou X. Sun J. Zhu B. Duan C. (2020). A New Self-Healing Hydrogel Containing hucMSC-Derived Exosomes Promotes Bone Regeneration. Front. Bioeng. Biotechnol. 8, 564731. 10.3389/fbioe.2020.564731 33042966 PMC7521201

[B88] Wen J. H. Vincent L. G. Fuhrmann A. Choi Y. S. Hribar K. C. Taylor-Weiner H. (2014). Interplay of Matrix Stiffness and Protein Tethering in Stem Cell Differentiation. Nat. Mater 13 (10), 979–987. 10.1038/nmat4051 25108614 PMC4172528

[B89] Wu G. Xu P. C. Wu P. Hu K. Sun Y. Cheng B. (2017). Advances in the Treatment of Rotator Cuff Lesions by Cytokines. Front. Biosci. (Landmark Ed. 22, 516–529. 10.2741/4499 27814629

[B90] Xie J. Li X. Lipner J. Manning C. N. Schwartz A. G. Thomopoulos S. (2010). "Aligned-to-random" Nanofiber Scaffolds for Mimicking the Structure of the Tendon-To-Bone Insertion Site. Nanoscale 2 (6), 923–926. 10.1039/c0nr00192a 20648290 PMC3609028

[B91] Yadav H. Nho S. Romeo A. MacGillivray J. D. (2009). Rotator Cuff Tears: Pathology and Repair. Knee Surg. Sports Traumatol. Arthrosc. 17 (4), 409–421. 10.1007/s00167-008-0686-8 19104772

[B92] Yang R. Li G. Zhuang C. Yu P. Ye T. Zhang Y. (2021). Gradient Bimetallic Ion-Based Hydrogels for Tissue Microstructure Reconstruction of Tendon-To-Bone Insertion. Sci. Adv. 7 (26). 10.1126/sciadv.abg3816 PMC8221628 34162547

[B93] Zhang Z.-Z. Zhou Y.-F. Li W.-P. Jiang C. Chen Z. Luo H. (2019). Local Administration of Magnesium Promotes Meniscal Healing through Homing of Endogenous Stem Cells: A Proof-Of-Concept Study. Am. J. Sports Med. 47 (4), 954–967. 10.1177/0363546518820076 30786213

[B94] Zhao D. Yu Z. Li Y. Wang Y. Li Q. Han D. (2020). GelMA Combined with Sustained Release of HUVECs Derived Exosomes for Promoting Cutaneous Wound Healing and Facilitating Skin Regeneration. J. Mol. Hist. 51 (3), 251–263. 10.1007/s10735-020-09877-6 32388839

[B95] Zhao S. Su W. Shah V. Hobson D. Yildirimer L. Yeung K. W. K. (2017). Biomaterials Based Strategies for Rotator Cuff Repair. Colloids Surfaces B Biointerfaces 157, 407–416. 10.1016/j.colsurfb.2017.06.004 28633121

